# The mango core quality non-destructive testing and comprehensive harvesting decision-making model based on portable near-infrared spectroscopy technology

**DOI:** 10.3389/fpls.2026.1772717

**Published:** 2026-03-20

**Authors:** Ruilong Hao, Zhe Wang, Jianhua Zhang, Xiwen Kang, Huiqin Ma, Guoping Yu

**Affiliations:** 1 National Nanfan Research Institute (Sanya), Chinese Academy of Agricultural Sciences, Sanya, China; 2 College of Horticulture, China Agricultural University, Beijing, China; 3 Weatherhead School of Management, Case Western Reserve University, Cleveland, OH, United States

**Keywords:** fruit firmness, mango, maturity, pH value, portable non-destructive testing

## Abstract

It is of great significance to detect the degree of mango maturity accurately and quickly in terms of timely harvesting according to market needs and building a differentiated post-harvest field sorting system. In this study, three main mango varieties—’Tainong’, ‘Guifei’, and ‘Jinhuang’—that originated in Hainan, China, were used as subjects. An H-100F portable near-infrared spectrometer was used to collect the absorption spectra between 650- and 950-nm wavelengths at 20 days before as well as 20 days after the commercial harvesting time. Four types of quality indicators, including fruit firmness, pH value, soluble solid content (SSC), and dry matter content (DMC), were measured in the laboratory to finally establish the non-destructive detection (NDT) and comprehensive harvesting decision-making model. As the results indicated, in the range of 650–950 nm, the average value of the spectrum absorbance for the three mango varieties decreased as the grade of maturity increased. The value change correlated significantly with fruit firmness, pH, SSC, and DMC (%). The Kennard–Stone algorithm method was used to divide the four quality indicators into correction sets and prediction sets. The original spectrum and the spectrum preprocessed using six methods, including multiplicative scatter correction, standard normal variate transform, Savitzky–Golay convolution smoothing, Savitzky–Golay convolution derivative, vector normalization, and maximum–minimum normalization, were then preferably selected to build a processing model for the four quality indicators of the three mango varieties. The test results showed that for all three mango varieties, both the revised standard error of mean squared root and the indicated standard error of mean squared root of the four quality indicators were less than 1.42, 0.62, 0.78, and 1.4, and 1.52, 0.38, 0.94, and 1.5, respectively. The accuracy rates of the model to test the grade of maturity for ‘Tainong’, ‘Guifei’, and ‘Jinhuang’ were 83%, 90%, and 81%, respectively. The above results indicated that the mango NDT model that was established on the portable near-infrared spectroscopy technology can reliably detect the fruit firmness, pH, SSC, and DMC of mangoes and can distinguish the grade of maturity for different mango varieties. The research results are of great significance to the decision-making of mango harvesting and the differentiated field sorting thereafter.

## 1 Introduction

Mango (*Mangifera indica* L.), subordinated to the Anacardiaceae *Mangifera*, is a tropical evergreen tree (Lebaka et al., 2021). China is the world’s second-largest mango producer and the largest mango consumer. However, compared to the most advanced mango producers in the world, China has poorer product quality. Hainan, as a major mango-producing region in China, has a planting area of 66,169 hectares, with an output of 830,800 tons in 2023. The main varieties in Hainan are ‘Tainong’, ‘Guifei’, ‘Jinhuang’, and ‘White Ivory’.

As a respiratory leaping fruit, the color of a mango’s skin does not change obviously before its supposed harvest time (Yashoda et al., 2005), and the quality of fruit appearance during the harvest period differs by varieties, ages, and origin (Maldonado-Celis et al., 2019). Therefore, harvest based on color, size, and fruit shape often leads to inaccurate timing that is either earlier or later than the perfect harvest time (Dick et al., 2009; Ernesto et al., 2018). An early harvest leads to the insufficient accumulation of soluble solid content (SSC) and dry matter content (DMC), resulting in insufficient flavor and low quality when the fruit matures thereafter. A late harvest leads to aging of pulp fiber, poor consumption quality, and short shelf life. The commercial value of mango products will be affected in either case (Joas et al., 2009; Joas et al., 2012; Gianguzzi et al., 2021). Therefore, a quick and accurate non-destructive detection (NDT) method is urgently needed to recognize the perfect harvest timing and enhance the differentiated field sorting.

In recent years, near-infrared (NI) spectroscopy (Matthieu et al., 2021), hyperspectral imaging (Mohd et al., 2014; Mishra et al., 2017), electronic nose (Ren et al., 2017), and other technologies have been tried for non-destructive intrinsic quality testing on fruits (Bessho et al., 2007). Among them, the near-infrared spectroscopy method, combined with computer technology and stoichiometry methods, is suitable for the measurement of physicochemical parameters of hydrogen-containing organic subjects (Bouveresse and Massart, 1996; Beringer et al., 2012). With characteristics of no sample preprocessing, non-destructive, fast detection, and no pollution (Celio, 2018; Anyidoho et al., 2020; Semyalo et al., 2024), this method has become an important technical method for non-destructive testing of agricultural products.

Near-infrared spectroscopy has been applied by many scholars to detect the intrinsic quality variations and the best harvest time during the fruit harvest period. Subedi used short-wave near-infrared spectroscopy to establish a partial least squares regression (PLSR) model and successfully detected the DMC of mango (Subedi and Walsh, 2011). From the similarity of B coefficients, this model can better verify the grade of maturity (R^2^ = 0.94, RMSE = 0.90). At the same time, near-infrared spectroscopy has also been applied to build detection models, such as those including mango starch content (Saranwong et al., 2003) and firmness (Valente et al., 2009), to determine the grade of maturity. However, the growth and development of maturity are characterized by various indexes including firmness, pH, SSC, and DMC (Serna-Escolano et al., 2024). The current portable non-destructive testing equipment has only one judgment index, which leads to poor application results. Some scholars have established a comprehensive evaluation index (CEI) of maturity according to the changes in physical and chemical properties of fruits, such as the Streif index, factor quality index (FQI), and simplified internal quality index (SIQI). The comprehensive evaluation index was used to classify the ripeness of fruits, and the problem of inconsistent fruit harvesting evaluation criteria was solved (Li et al., 2021). This study established a comprehensive evaluation index F based on fruit firmness, pH, SSC, and DMC that was derived from mangoes harvested between 20 days before and 20 days after commercial harvest time. It showed significant reference value for both the non-destructive dynamic monitoring of mango quality and the scientific decision-making of harvest time. At the same time, the portable feature and field-applicable scenarios are of great significance to the application and promotion of the detection technology.

## 2 Materials and methods

### 2.1 Materials

The mango samples were collected from trees that were located in Tianya District, Sanya City, Hainan Province, and that were approximately 20 days before commercial harvest time. Samples were collected randomly every 4 days. Each of 25 samples from ‘Tainong’, ‘Guifei’, and ‘Jinhuang’ was randomly selected, and each variety of samples was of a similar size and non-destructive and had no pests. Samples were delivered to the laboratory of Sanya Research Institute of China Agricultural University within 1 hour after being collected. Then, the near-infrared absorption spectrum, fruit firmness, pH, SSC, and DMC were measured. Sampling continued until 20 days after the commercial grade of maturity was reached. ‘Tainong’ was sampled 11 times, ‘Guifei’ was sampled 10 times, and ‘Jinhuang’ was sampled 10 times. The total sample size was 775.

### 2.2 Methods

#### 2.2.1 Determination of near-infrared spectrum, firmness, pH, SSC, and DMC

The equipment used to test the near-infrared spectrum was an H-100F portable non-destructive test instrument with the integration time set to 2,000 ms. The spectral information collected was at the fixed point of the middle-upper part of the back of the mango samples in the 650–950-nm band. Then, after removing the upper peel on the back of the mango, the firmness was tested at 15 mm/s using a probe with a diameter of 8.5 mm (Agrosta Instrument, Seine-Maritime, France). The mango was cut along the core, a 5 cm * 3 cm * 1 mm slice was obtained near the outlet, the slice was weighed electronically (Sadis BSA124S-CW, Sartorius AG, Gottingen, Germany) and placed in a 70 °C blast drying box (DHG-9053A, Jiecheng Instrument, Shanghai, China) for 28 h until the weight stopped changing, and DMC was calculated. At the same time, the remaining pulp was squeezed, and SSC was measured using a portable digital display glycometer (AP-S50, Airipu Instrument, Zhejiang China); pH was tested using a pH meter (PHS-3E, INESA (Group), Shanghai, China).

#### 2.2.2 Establishment and quality evaluation of NIR NDT model

The mean near-infrared spectrum absorbance values of the 1st, 3rd, 5th, 7th, 9th, and 11th batches were calculated; the mean fruit firmness, pH, SSC, and DMC of each batch were calculated for all three varieties.

The outlier test of the spectral data was conducted. After removing any outliers, the Kennard–Stone algorithm (Pan, 2011) was used to divide the measured data of various mango physiological indicators into a correction set and verification set at a ratio of 3:1. Models were built using the correction set data, and model performance was verified using verification set data. Established models were preprocessed using methods including multiplicative scatter correction (MSC) (Geladi et al., 1985), the standard normal variate transform (SNV) (Jie et al., 2010), Savitzky–Golay (S-G) convolution smoothing (Seah et al., 2000), Savitzky–Golay (S-G) convolution derivative, vector normalization, and maximum–minimum homing method (Gajera et al., 2016). Then, a partial least squares non-destructive testing model was built using Unscrambler. The partial least squares model is expressed by Equation 1:

(1) Y=bX+e

In Formula 1, b is the vector of the regression coefficient, and e is the model residual.

The optimal model was selected by combining the correlation coefficient of correction (Rc), root mean square error of correction (RMSEC), correlation coefficient of prediction (Rp), and root mean square error of prediction (RMSEP) (Wei et al., 2020).

#### 2.2.3 Determination of mango maturity

This study used SPSS Statistics 26 to test Pearson’s correlation of firmness, pH value, SSC, DMC, and actual collecting time, and to analyze the correlation between the four indicators and the grade of maturity.

This study used principal component analysis (PCA) to analyze the distribution of the four indexes’ values (Shlens, 2014). Regression was used to calculate the score coefficient matrix (Bailey et al., 1963), and then the expression was constructed to reflect the comprehensive evaluation index F (Equation 2).

(2) $$\text{F}=\text{a}*{\text{Zx}}_{1}+\text{b}*{\text{Zx}}_{2}+\text{c}*{\text{Zx}}_{3}+\text{d}*{\text{Zx}}_{4}$$

In Formula 2, Zx_1_, Zx_2_, Zx_3_, and Zx_4_ stand for fruit firmness, pH, SSC, and DMC, respectively.

The grade of maturity of mangoes that just reached the commercial harvest standard was defined as the 6.5th grade, after which the 0.5 level was added every 8 days beyond the commercial harvest standard, recorded as the 7th, 7.5th, 8th, 8.5th, and 9th grades. The F value of each sample was calculated using the constructed formula; then, the F-value results were plotted using box plots for statistical analysis purposes.

## 3 Results

### 3.1 NIR spectral change during maturity

**Figure 1:**
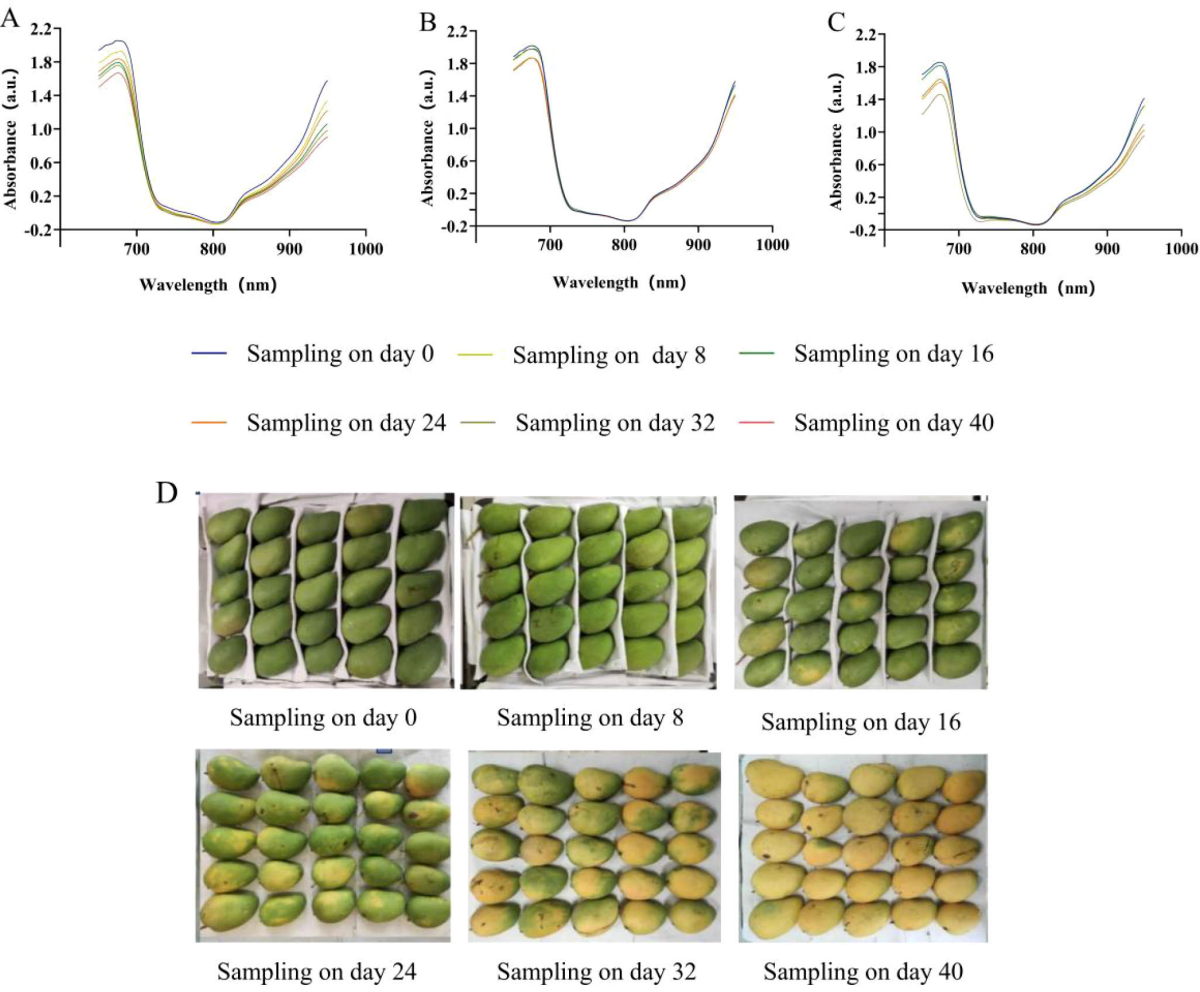
Average absorbance of the three mango varieties at different levels of maturity. **(A)** Tainong mango, **(B)** Guifei mango, and **(C)** Jinhuang mango. **(D)** The mango at harvest and different maturity stages.

The average spectrum absorbance value of the three mango varieties decreased as the grade of maturity increased (**Figure 1**). It deviated the most in the range of 650–700 nm. In the range of 704–800 nm, the spectrum absorbance value first decreased rapidly and then slowly to a trough value at 805 nm, where little difference was shown in the spectrum absorbance values for different grades of maturity. When the wavelength was greater than 840 nm, the spectrum absorbance values of ‘Tainong’ and ‘Jinhuang’ increased continuously, and the differences among different grades of maturity were obvious. However, the spectrum absorbance value showed a smaller difference among different grades of maturities for ‘Guifei’ compared to other varieties (**Figure 1**).

The gradual decrease in the mean spectrum absorbance value was due to the increasing clearance of the cells and transparency inside the fruit associated with increasing maturity. The trough value near 805 nm was mainly associated with the multiplied frequency and flexible vibration of the NH_2_ key (Polesello et al., 1983). The rapid increase in the spectrum absorbance value in the range of 900–950 nm was due to the increasing dry matter content and large accumulation of C–H, C–H_2_, C–H_3_, and OH as the grade of maturity increased (Rambla et al., 1997; Theanjumpol et al., 2013).

### 3.2 Core quality change during maturity

**Figure 2:**
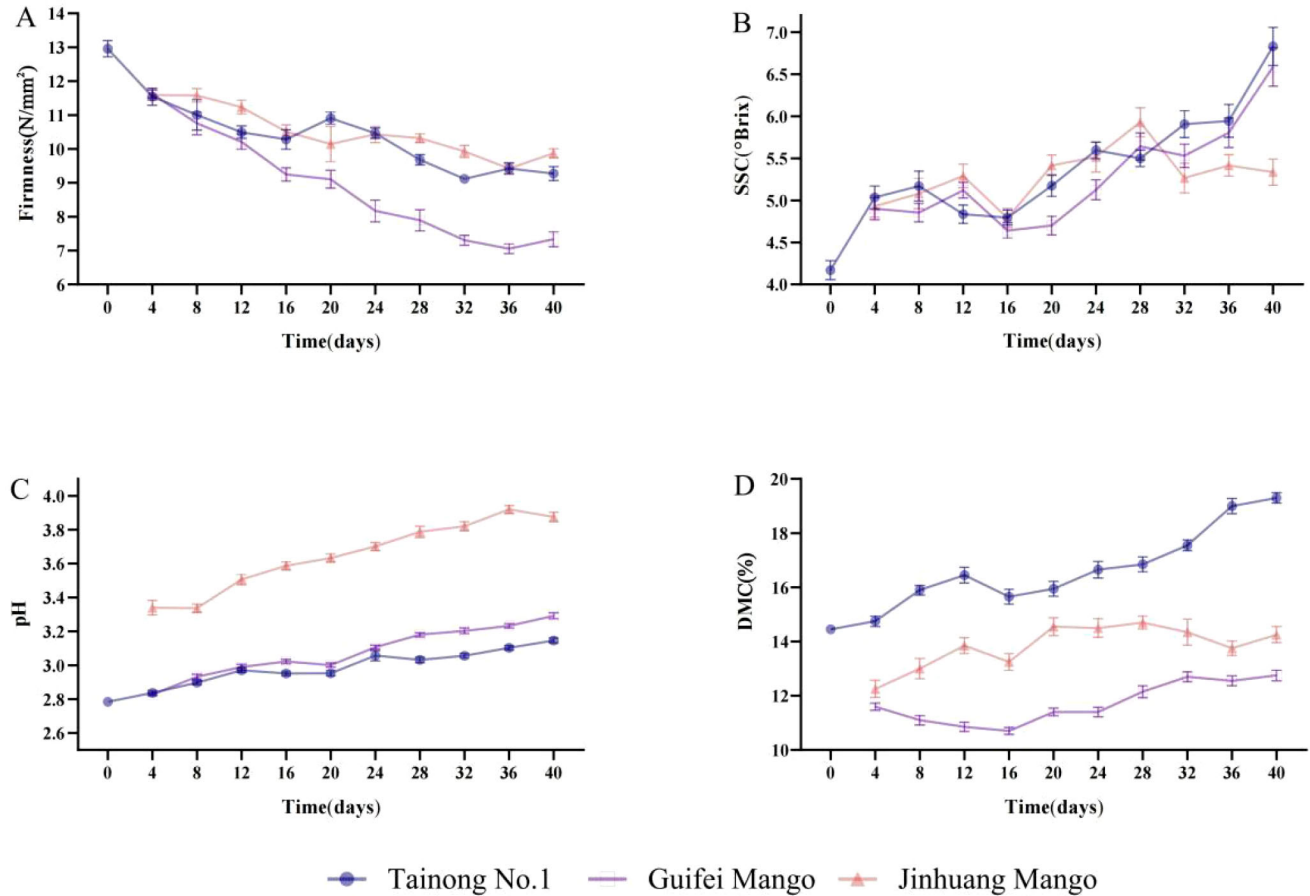
Correlation between the four quality indexes and the different levels of maturity. **(A)** Firmness, **(B)** pH, **(C)** SSC, and **(D)** DMC. SSC, soluble solid content; DMC, dry matter content.

The change in fruit firmness of the three varieties is shown in **Figure 2A**. The fruit firmness of ‘Tainong’, ‘Guifei’, and ‘Jinhuang’ decreased from 13, 11.6, and 11.6 N/mm^2^ to 9.3, 9.9, and 7.3 N/mm^2^ after 40 days, respectively. On days 20–24, the values peaked at 10.9 and 10.4 N/mm^2^ of ‘Tainong’ and ‘Jinhuang’, respectively. On days 32–36, the values peaked at 9.1, 9.4, and 7.1 N/mm^2^ of ‘Tainong’, ‘Guifei’, and ‘Jinhuang’, respectively. With increasing maturity, the pectin broke down under the action of pectinase, resulting in cell-free and reduced fruit firmness (Parre and Geitmann, 2005). However, the value of firmness was also susceptible to rainy weather, resulting in a rebound phenomenon.

The change in pH value of the three mango varieties is shown in **Figure 2B**. The pH values of ‘Tainong’, ‘Guifei’, and ‘Jinhuang’ increased from 2.78, 2.83, and 3.34 to 3.15, 3.29, and 3.88, respectively. The difference in pH values between ‘Tainong’ and ‘Guifei’ in the same period was small; the pH values of matured ‘Jinhuang’ were at a higher level compared to those of the immature ones. With the increase in grade of maturity, the pH values of the three varieties increased steadily and were not affected by weather changes.

The change in SSC during the maturity period of mango is shown in **Figure 2C**. The SSC of ‘Tainong’, ‘Guifei’, and ‘Jinhuang’ increased from 4.2°Brix, 4.9°Brix, and 4.9°Brix to 6.8°Brix, 5.3°Brix, and 6.6°Brix, respectively. On days 8–12, the SSC of the three varieties decreased rapidly after the peak and then recovered rapidly after the trough value on day 16. As the maturity duration increased, the change in SSC generally increased, but it was susceptible to weather changes. The suitable weather led to rapidly accumulated SSC values as fruit maturity increased, while prolonged rainy weather prevented SSC increases.

The change in DMC of the three varieties is shown in **Figure 2D**. The DMC of ‘Tainong’, ‘Guifei’, and ‘Jinhuang’ increased from 14%, 12%, and 12% to 19%, 13%, and 14%, respectively. Tainong’s DMC had the biggest increase at 36%. The DMC of ‘Guifei’ remained unchanged after it rapidly increased to 13% on day 32. The DMC of ‘Jinhuang’ stabilized at a level above 14% after the valley value on day 16. As the maturity duration increased, the DMC of the three varieties showed an increasing trend. However, it was susceptible to weather changes, which led to the DMC fluctuating.

### 3.3 NIR sample set division

The distribution of range, mean, standard deviation, and coefficient of variation derived from fruit firmness, pH, SSC, and DMC in the calibration sets and validation sets is shown in **Table 1**. The coefficient of variation and the data dispersion of the 24 sets were small, which ensured the accuracy of model building. The overall sample data conform to the normal distribution, which is suitable for establishing the NDT model using the partial least squares method.

**Table 1:** Sample summary characteristics of the four quality indexes on three mango varieties.

Variety	Classification	Index	Number of samples	Maximum value	Minimum value	Extreme value	Mean
Tainong mango	Correction	Firmness (N/mm^2^)	162	7.1	14.7	7.6	10.42
		pH	162	2.7	3.28	0.58	2.98
		SSC (°Brix)	161	3.4	8.4	5	5.46
		DMC	162	0.131	0.211	0.081	0.166
	Prediction	Firmness (N/mm^2^)	54	8	15.3	7.3	10.5
		pH	53	2.73	3.21	0.48	2.98
		SSC (°Brix)	53	3.6	9.7	6.1	5.52
		DMC (%)	54	13.6	21.2	7.6	16.7
Guifei mango	Correction	Firmness (N/mm^2^)	149	4.4	14	9.6	8.86
		pH	148	2.76	3.4	0.64	3.08
		SSC (°Brix)	147	4.1	8.5	4.4	5.52
		DMC	147	0.098	0.143	0.046	0.117
	Prediction	Firmness (N/mm^2^)	49	5.7	12.8	7.1	8.88
		pH	49	2.77	3.41	0.64	3.08
		SSC	196	4.1	9.2	5.1	5.54
		DMC (%)	49	9.8	14.4	4.6	11.8
Jinhuang mango	Correction	Firmness (N/mm^2^)	149	8.1	13.3	5.2	10.53
		pH	147	3.03	4.05	1.02	3.65
		SSC (°Brix)	148	4	7.1	3.1	5.29
		DMC	147	0.099	0.183	0.084	0.138
	Prediction	Firmness (N/mm^2^)	50	8.5	13.3	4.8	10.59
		pH	48	3.09	4.05	0.96	3.67
		SSC (°Brix)	48	4.1	7.1	3	5.3
		DMC (%)	49	10.3	18.5	8.2	13.9

SSC, soluble solid content; DMC, dry matter content.

### 3.4 Preprocessing of near-infrared spectrum and the establishment of partial least squares model

**Figure 3:**
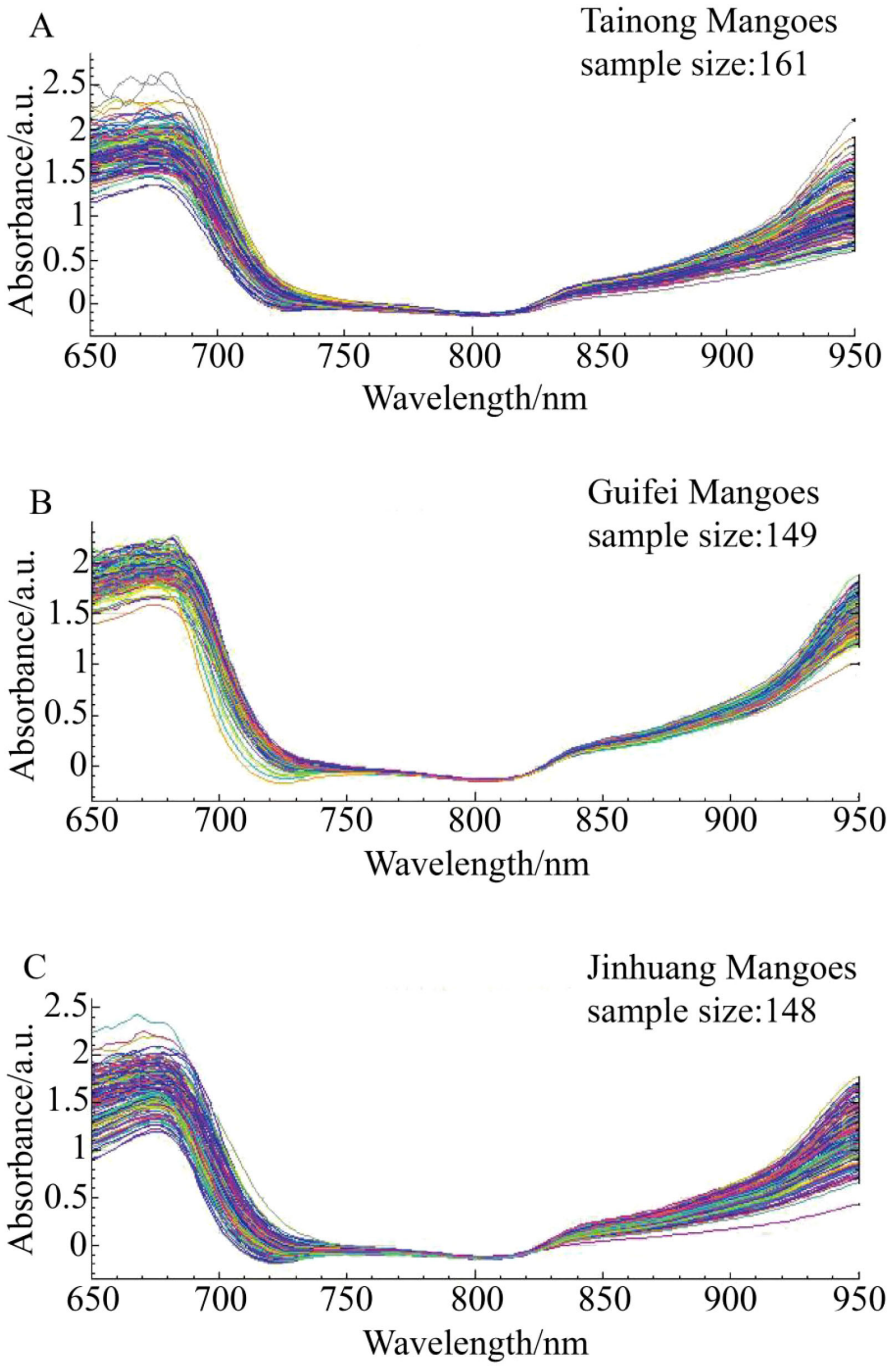
The spectrum distribution characteristics of three mango varieties in the modeling set across the entire sampling period. **(A)** Tainong Mangoes, **(B)** Guifei Mangoes, **(C)** Jinhuang Mangoes.

**Figure 4:**
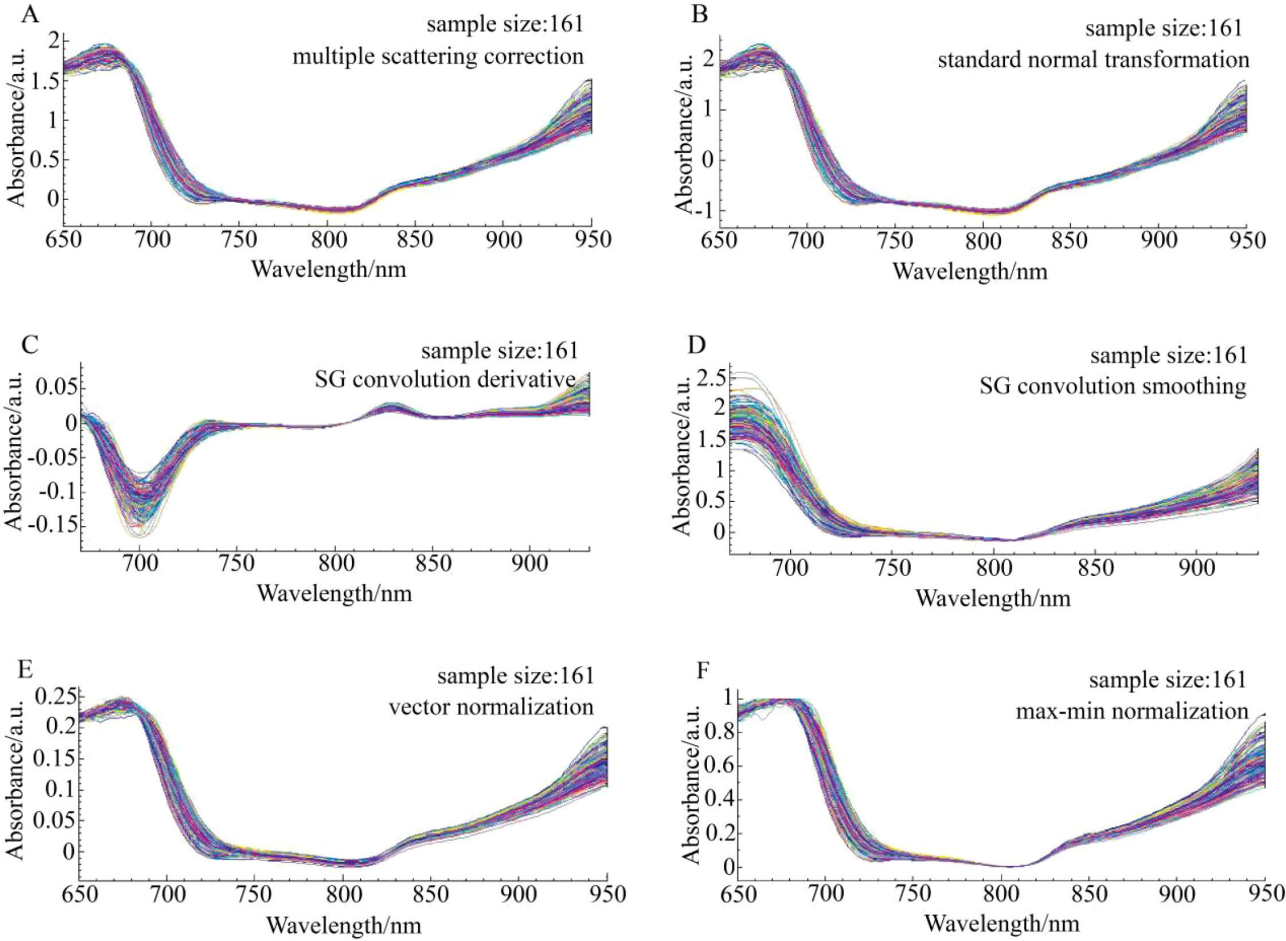
The spectrum distribution characteristics of Tainong mangoes in the modeling set across the entire sampling period under six preprocessing methods. **(A)** Multiple scattering correction (MSC), **(B)** Standard normal transformation (SNV), **(C)** SG convolution derivative, **(D)** SG convolution smoothing, **(E)** Vector normalization, **(F)** Max-min normalization.

**Figure 5:**
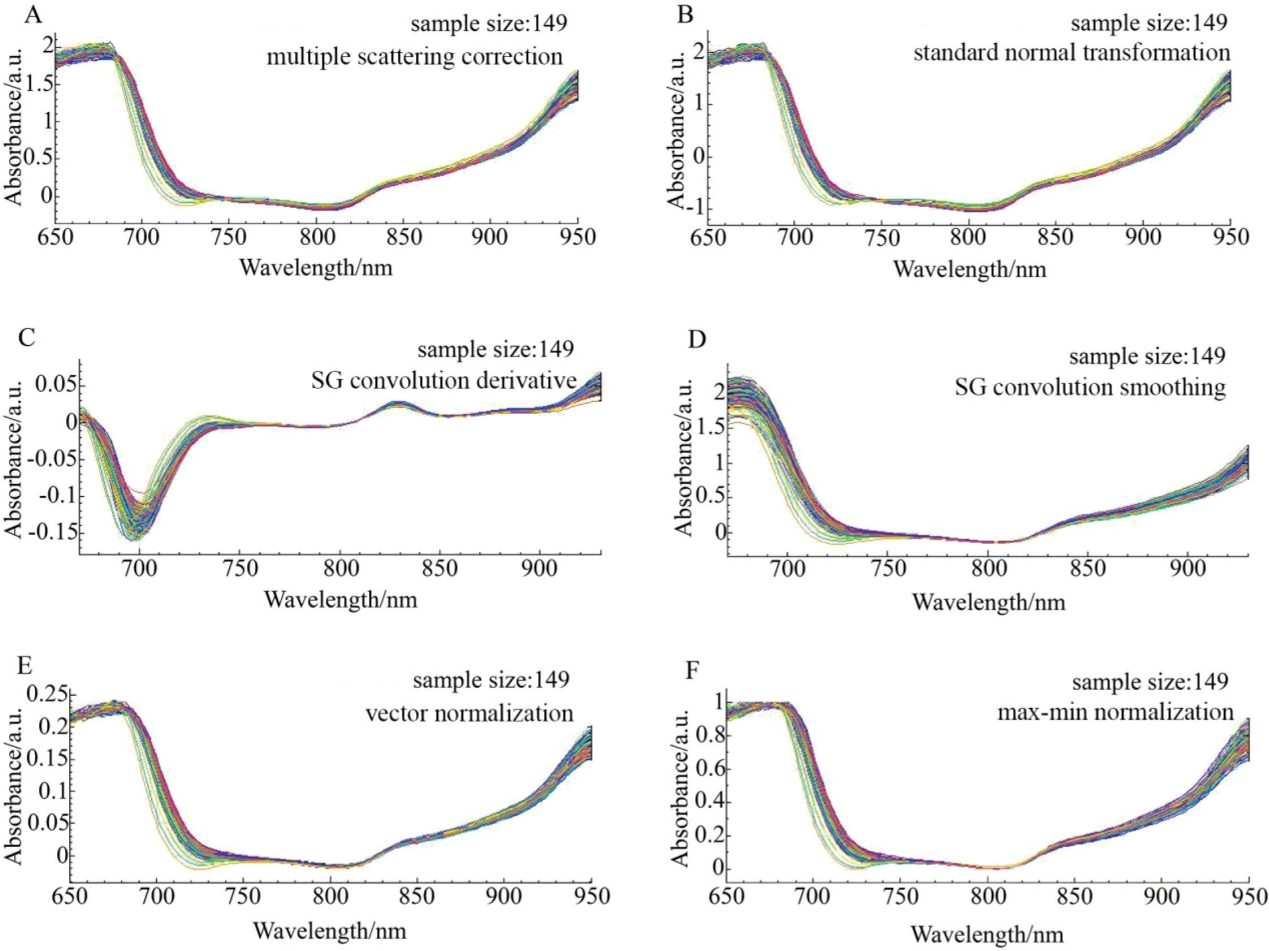
The spectrum distribution characteristics of Guifei mangoes in the modeling set across the entire sampling period under six preprocessing methods. **(A)** Multiple scattering correction (MSC), **(B)** Standard normal transformation (SNV), **(C)** SG convolution derivative, **(D)** SG convolution smoothing, **(E)** Vector normalization, **(F)** Max-min normalization.

**Figure 6:**
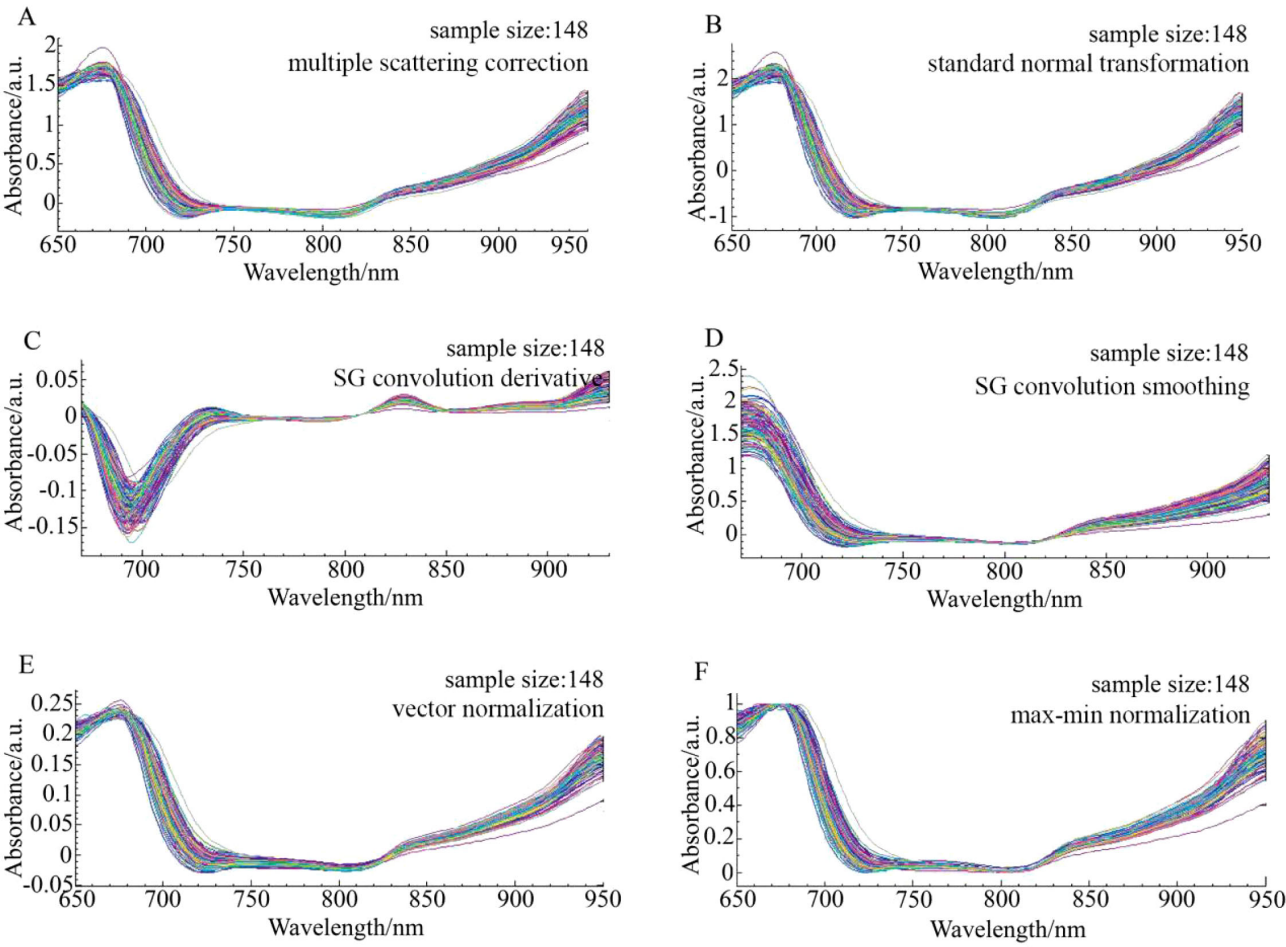
The spectrum distribution characteristics of Jinhuang mangoes in the modeling set across the entire sampling period under six preprocessing methods. **(A)** Multiple scattering correction (MSC), **(B)** Standard normal transformation (SNV), **(C)** SG convolution derivative, **(D)** SG convolution smoothing, **(E)** Vector normalization, **(F)** Max-min normalization.

The original spectra of ‘Tainong’, ‘Guifei’, and ‘Jinhuang’ were preprocessed using six algorithms, as shown in **Figures 3**–**6**, respectively. The original spectrum was preprocessed using MSC or SNV, which eliminated the scattering effect caused by uneven tissue in the sample, resulting in the spectra from 650 to 700 nm showing a high degree of polymerization (**Figures 4A, B**). After the SG convolution derivative method, new characteristic peaks appeared near 700 and 730 nm, and the tiny characteristic peaks near the wavelength of 830 nm were further enlarged (**Figure 4C**). After the SG convolution smoothing method, the random noise in the 650–750- and 850–900-nm bands was obviously eliminated, making the spectral curve smoother, but also removing some details in the spectrum, leading to the weakening of the overall characteristics of the spectrum (**Figure 4D**). After the vector normalization, the overall spectrum was relatively polymetric, and the trending was strengthened. However, the averaging method weakened the aggregation characteristics of the spectrum in the 750–850-nm wavelength range that had strong polymerization before (**Figure 4E**). After the maximum–minimum normalization, the spectrum was closely aggregated in the area where the data were concentrated, but the maximum and minimum values of the data were unstable, resulting in worse aggregation after 850 nm (**Figure 4F**).

The four indicators of firmness, pH, SSC, and DMC under six different algorithms were tested using a partial least squares model (**Table 2**). After the raw data of firmness levels of ‘Tainong’ and ‘Guifei’ were processed using the six algorithms, the overall optimization degree of the model was not high. Therefore, raw data can be preferred to build the optimal model. The optimal model to process the raw data of ‘Jinhuang’ firmness levels was standard normal transformation. The optimal models to process the raw data of ‘Tainong’, ‘Guifei’, and ‘Jinhuang’ pH values were vector normalization, multivariate scattering correction, and vector normalization, respectively. The optimal models to process the raw data of ‘Tainong’, ‘Guifei’, and ‘Jinhuang’ SSCs were maximum–minimum normalization, convolution derivatives, and standard normal transformation, respectively. The optimal models to process the raw data of ‘Tainong’, ‘Guifei’, and ‘Jinhuang’ DMCs were vector small normalization, convolution smoothing, and convolution derivatives, respectively.

**Table 2:** Quality test of the non-destructive testing model under different preprocessing methods.

Parameter	Preprocessing method	R_C_ (T/G/J)			RMSEC (T/G/J)			R_P_ (T/G/J)			RMSEP (T/G/J)		
Firmness	Original spectrum	0.63	0.76	0.85	1.267	1.424	0.891	0.55	0.65	0.78	1.359	1.518	0.847
	Maximum–minimum normalization	0.50	0.63	0.54	1.282	1.466	0.9	0.43	0.53	0.58	1.374	1.57	0.857
	SG convolution derivative	0.50	0.65	0.54	1.291	1.437	0.9	0.47	0.58	0.60	1.345	1.452	0.846
	Multiplicative scatter correction	0.50	0.62	0.55	1.285	1.484	0.893	0.45	0.53	0.58	1.360	1.565	0.857
pH	Original spectrum	0.67	0.82	0.77	0.093	0.090	0.153	0.67	0.70	0.72	0.085	0.108	0.167
	Vector normalization	0.79	0.71	0.83	0.095	0.110	0.161	0.72	0.53	0.75	0.082	0.13	0.147
	Multiple scattering correction	0.62	0.89	0.73	0.098	0.092	0.164	0.65	0.81	0.74	0.088	0.108	0.16
	Standard Normal Variate transform	0.63	0.62	0.74	0.097	0.122	0.161	0.64	0.38	0.78	0.088	0.143	0.147
SSC	Original spectrum	0.56	0.66	0.63	0.779	0.730	0.585	0.50	0.62	0.51	0.886	0.84	0.606
	Maximum–minimum normalization	0.66	0.49	0.64	0.779	0.840	0.578	0.57	0.49	0.47	0.886	0.94	0.626
	SG convolution derivative	0.54	0.67	0.57	0.787	0.720	0.612	0.45	0.61	0.60	0.916	0.85	0.568
	Standard Normal Variate transform	0.55	0.64	0.68	0.781	0.750	0.551	0.53	0.43	0.60	0.927	0.96	0.606
DMC	Original spectrum	0.61	0.69	0.67	0.015	0.007	0.012	0.53	0.45	0.69	0.015	0.0095	0.012
	SG convolution derivative	0.45	0.79	0.75	0.016	0.006	0.011	0.50	0.67	0.75	0.015	0.0076	0.011
	SG convolution smoothing	0.38	0.81	0.66	0.017	0.006	0.012	0.48	0.74	0.64	0.016	0.007	0.013
	Vector normalization	0.75	0.56	0.71	0.014	0.008	0.011	0.66	0.40	0.69	0.015	0.010	0.012

T, Tainong mango; G, Guifei mango; J, Jinhuang mango; SSC, soluble solid content; DMC, dry matter content.

### 3.5 Model quality evaluation

**Figure 7:**
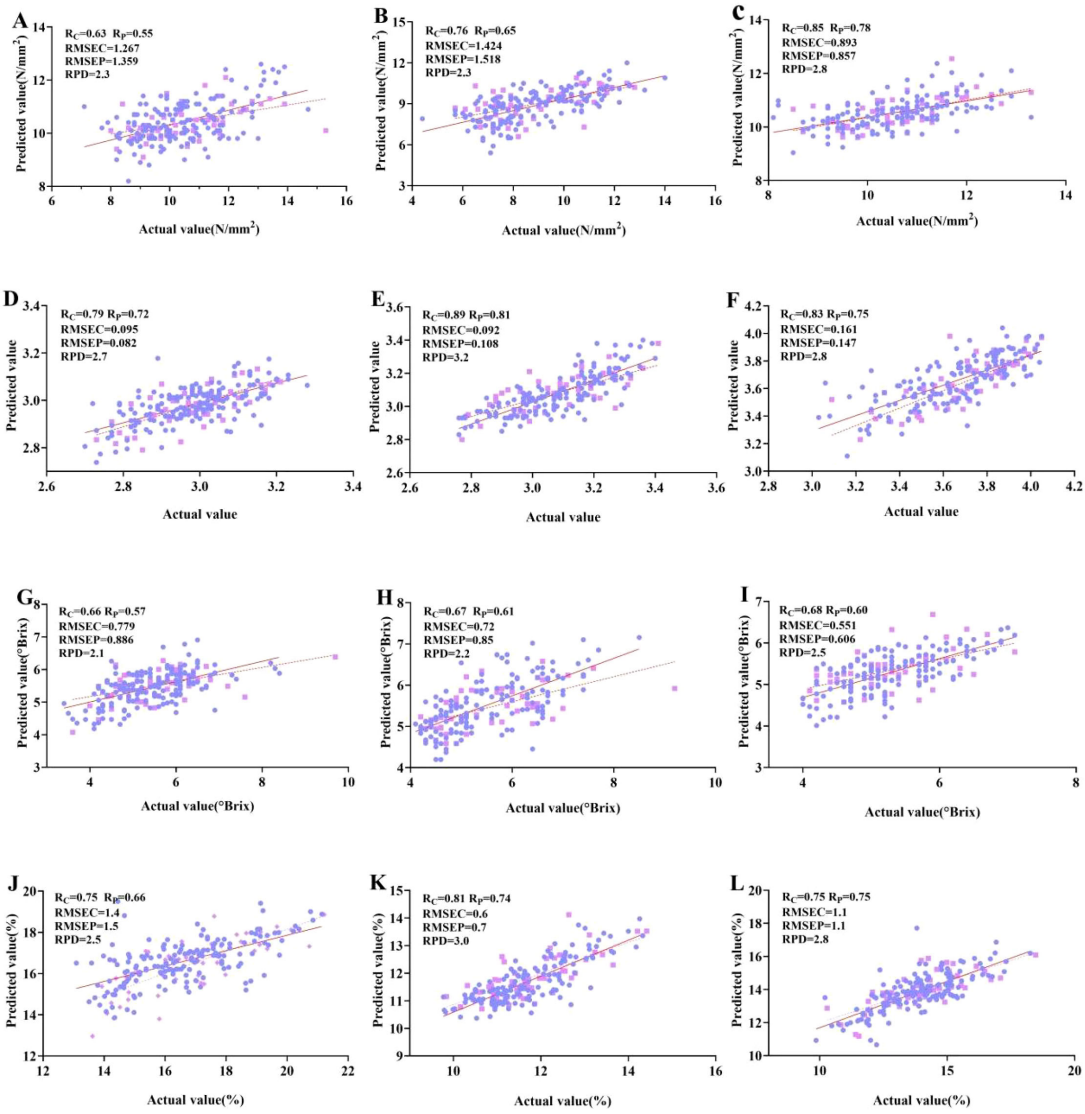
Fitting scatter plots of the four indexes of the three mango varieties under optimal PLS model. **(A)** Firmness of Tainong mango. **(B)** Firmness of Guifei mango. **(C)** Firmness of Jinhuang mango. **(D)** pH of Tainong mango. **(E)** pH of Guifei mango. **(F)** pH of Jinhuang mango. **(G)** SSC of Tainong mango. **(H)** SSC of Guifei mango. **(I)** SSC of Jinhuang mango. **(J)** DMC of Tainong mango. **(K)** DMC of Guifei mango. **(L)** DMC of Jinhuang mango. PLS, partial least squares; SSC, soluble solid content; DMC, dry matter content.

The scatter fitting results of the measured and indicated values of the fruit firmness models of ‘Tainong’, ‘Guifei’, and ‘Jinhuang’ are shown in **Figures 7A–C**. The indicated firmness value of ‘Tainong’ was mainly concentrated in the range of 9–12 N/mm^2^, within which the deviation degree of the indicated value was small, and the accuracy of the model was justified. However, due to the small number of high firmness samples, the dispersion degree of the indicated value was large in the range greater than 12 N/mm^2^, and the error degree of the indicated results was large. The number of such samples needed to be increased to further optimize the model. For ‘Guifei’ and ‘Jinhuang’, the measured firmness values and indicated values were evenly distributed on both sides of the fitting line, indicating that the model had good detection ability.

The scatter fitting results of the measured pH values and the indicated pH values of ‘Tainong’, ‘Guifei’, and ‘Jinhuang’ are shown in **Figures 7D–F**. The scatter of the model results of the three mango varieties was evenly distributed across the regions and was closely aggregated on both sides of the fitted curve, indicating that the model had a good ability to detect the pH value.

The scatter fitting results of the measured values and indicated SSC values of ‘Tainong’, ‘Guifei’, and ‘Jinhuang’ are shown in **Figures 7G–I**. The measured values and the indicated values established by the raw data of ‘Tainong’ were evenly distributed on both sides of the fitted curve. Overall, the model had a strong detection ability. However, there were fewer mango samples in the higher SSC interval, so the detection accuracy with SSC greater than 8°Brix was slightly lower. The SSC model of ‘Guifei’ had stable detection ability in the 4–7°Brix interval. However, it had less scatter in the 7–9°Brix interval and a relatively narrow detection range, which can be further optimized. The SSC model of ‘Jinhuang’ was evenly distributed in the whole region and closely arranged on both sides of the fitted curve, indicating that the model had good detection ability.

The scatter fitting results of the measured values and indicated DMC values of ‘Tainong’, ‘Guifei’, and ‘Jinhuang’ are shown in **Figures 7J–L**. The DMC scatter of ‘Tainong’ was distributed across the regions, indicating that the model had the power to detect the different DMC values of mango samples. There were some scatter points with a large deviation degree in section 0.13–0.15. This was because the mango samples in this section had a lower degree of maturity, thicker skin, and higher firmness. This led to the internal spectral information of the mango not easily collected using the near-infrared spectrometer. The detection ability can be improved by optimizing the light source of the equipment. The DMC scatter of both ‘Guifei’ and ‘Jinhuang’ had a relatively high fitting degree, and the number and degree of scatter deviations were within the error range, indicating that the model had good detection ability.

### 3.6 Correlation analysis of mango quality indicators

The correlation between the four indicators (fruit firmness, pH, SSC, and DMC) and the ripening time of the three mango varieties is shown in **Table 3**. The fruit firmness, pH, SSC, and DMC of Tainong and Guifei were all extremely significantly correlated with the ripening time. The fruit firmness and pH value of ‘Jinhuang’ mango were extremely significantly correlated with ripening time, and SSC and DMC were significantly correlated with ripening time. They also showed significant correlation between fruit firmness and pH, fruit firmness and SSC, fruit firmness and DMC, SSC and pH, SSC and DMC, and pH and DMC. The results showed that the value changes of the four indicators were not only affected by the maturity time but also correlated with each other. The establishment of a comprehensive evaluation index based on the above four quality indicators can better evaluate the degree of mango maturity.

**Table 3:** Pearson’s correlation analysis between the four quality indexes and maturity time.

Variety		Firmness	pH	SSC	DMC	Time
Tainong mango	Firmness	1	−0.907^**^	−0.815^**^	−0.920^**^	−0.901^**^
	pH		1	0.894^**^	0.966^**^	0.957^**^
	SSC			1	0.880^**^	0.901^**^
	DMC				1	0.931^**^
	Time					1
Guifei mango	Firmness	1	−0.969^**^	−0.697^*^	−0.621	−0.974^**^
	pH		1	0.834^**^	0.679^*^	0.982^**^
	SSC			1	0.808^**^	0.823^**^
	DMC				1	0.719^*^
	Time					1
Jinhuang mango	Firmness	1	−0.952^**^	−0.454	−0.584	−0.919^**^
	pH		1	0.578	0.689^*^	0.978^**^
	SSC			1	0.887^**^	0.537
	DMC				1	0.639^*^
	Time					1

SSC, soluble solid content; DMC, dry matter content.

^*^The correlation was significant at level of 0.05 (two-tailed).

^**^The correlation was significant at level of 0.01 (two-tailed).

### 3.7 The comprehensive mango harvesting decision-making model

**Figure 8:**
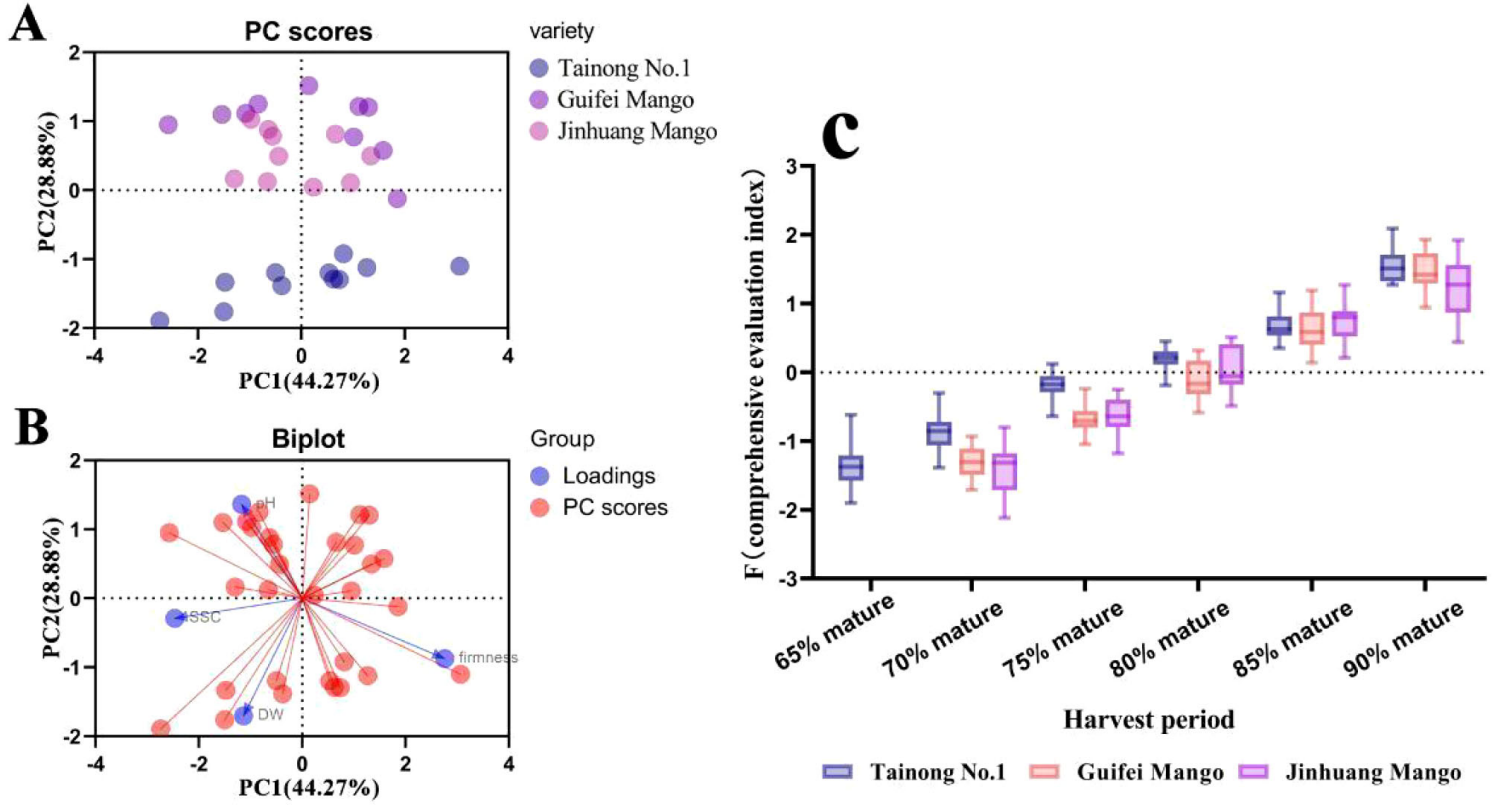
Maturity identification of the three mango varieties. **(A)** Principal component scores in principal component analysis. **(B)** Principal component biplots in principal component analysis. **(C)** The maturity of three mango varieties at different sampling points.

As the results of the principal components for the maturity degrees of the three mango varieties show in **Figure 8**, PC1 and PC2 had an interpretation rate of 44.27% and 28.88%, respectively, with an overall interpretation rate of 73.15%. Mango samples with different maturities were evenly distributed within the 95% confidence interval (**Figure 8A**). In terms of PC2, ‘Tainong’ was obviously different from ‘Guifei’ and ‘Jinhuang’, while the difference between ‘Guifei’ and ‘Jinhuang’ was small. As the biplot shows, the variance of the four mango quality indicators in terms of PC1 and PC2 was similar, but the distribution and direction of the four indicators in the graph were significantly different from each other: SSC and DMC were significantly positively correlated, while firmness and pH were significantly negatively correlated (**Figure 8B**). The four quality indicators were all principal components for interpreting the degree of maturity, and they had different effects on the maturity degree judgment.

Using the comprehensive evaluation index F to evaluate and validate the harvesting prediction results, the maturity discrimination results for mangoes are shown in **Figure 8C**: the accuracy rates for ‘Tainong’ were 85%, 85%, 80%, 70%, 85%, and 100% at the 6.5th, 7th, 7.5th, 8th, 8.5th, and 9th grades, respectively, and the overall accuracy rate was 83%. The accuracy rates for ‘Guifei’ were 100%, 80%, 85%, 90%, and 90% at the 7th, 7.5th, 8th, 8.5th, and 9th grades, respectively, and the overall accuracy rate was 90%. The accuracy rates for ‘Jinhuang’ were 85%, 95%, 80%, 70%, and 75% at the 7th, 7.5th, 8th, 8.5th, and 9th grades, respectively, and the overall accuracy rate was 81%. The accuracy rates were higher at the 7th and 7.5th grades than those at the 8th, 8.5th, and 9th grades, which indicates that the model should be further optimized for the decision-making at these maturity grades.

## 4 Discussion

Leveraging portable near-infrared spectroscopy (NIRS), this work established non-destructive predictive models for key internal quality attributes of mango, including firmness, pH, SSC, and DMC, and further integrated these outputs into a comprehensive harvesting decision model for maturity discrimination. While the overall performance supports the feasibility of portable NIRS for in-field maturity assessment, several considerations remain critical for subsequent methodological refinement and broader deployment.

### 4.1 Validity and robustness of the modeling framework

The portable instrumentation and chemometric workflow employed here enable rapid, non-destructive acquisition of intrinsic quality information, which is particularly advantageous for repeated monitoring across the commercial harvest window. The consistent performance observed across calibration and independent prediction sets suggests that the models captured chemically meaningful variation associated with ripening and were sufficiently stable for pre- and post-harvest window quality tracking (Arendse et al., 2018). From an operational perspective, such robustness is a prerequisite for supporting data-driven harvest scheduling and precision orchard management (Freitas et al., 2022). Nevertheless, further enlargement and diversification of the sample base—especially across seasons and orchards—will be essential to strengthen generalizability and to mitigate the risk of overfitting to the current sampling domain.

### 4.2 Transferability and scalability across cultivars and production regions

The present models were optimized for the three dominant cultivars cultivated in Hainan and achieved high discrimination accuracy under the studied conditions. However, spectral responses and physicochemical trajectories can vary substantially across cultivars and agro-ecological contexts (Mishra et al., 2017). A practical pathway for model scaling is therefore the systematic acquisition of paired spectral–physicochemical datasets from additional representative cultivars, followed by the construction of a structured model library using a consistent preprocessing and calibration protocol. Such a “single-instrument, multi-model” strategy can preserve predictive fidelity while expanding applicability, thereby improving the utility of the device and facilitating wider adoption and standardization in commercial settings.

### 4.3 Implications for industrial adoption and downstream value chains

Beyond harvest-time determination, the integrated decision model has potential value for post-harvest grading (El-Mesery et al., 2019), flavor-oriented sorting, and quality traceability throughout the supply chain (Pu et al., 2021). Importantly, the modeling outputs are amenable to integration into smart-agriculture platforms, enabling real-time data acquisition, cloud-based analytics, and decision feedback loops, which collectively support the digitalization and refinement of mango production and circulation systems. In addition, the workflow proposed here may be extendable to other climacteric fruits (e.g., banana and kiwifruit) (Nordey et al., 2016), offering a transferable methodological reference for non-destructive maturity evaluation under field-relevant constraints (Ecarnot et al., 2013; Pereira et al., 2018; Altieri et al., 2025).

Overall, the proposed models exhibit both methodological novelty and practical relevance, providing a technically viable route toward intelligent maturity management in mango production. Future work should prioritize algorithmic optimization, increased sample heterogeneity, and enhanced field adaptability of the portable device to promote broader application across production, post-harvest handling, and distribution chains.

## Data Availability

The original contributions presented in the study are included in the article/supplementary material. Further inquiries can be directed to the corresponding authors.
